# Intradiscal Gelified Ethanol Nucleolysis versus Endoscopic Surgery for Lumbar Disc Herniation Radiculopathy

**DOI:** 10.3390/diagnostics13132164

**Published:** 2023-06-25

**Authors:** Christos Gogos, Dimitrios K. Filippiadis, Georgios Velonakis, Nikolaos Kelekis, Panayiotis Papagelopoulos, Alexis Kelekis

**Affiliations:** 1 Neurosurgery Clinic, General Hospital “Asklepieio”, 16673 Athens, Greece; neurocare.gogos60@gmail.com; 2 2nd Department of Radiology, University General Hospital “ATTIKON”, Medical School, National and Kapodistrian University of Athens, 12462 Athens, Greece; giorvelonakis@gmail.com (G.V.); kelnik@med.uoa.gr (N.K.); akelekis@med.uoa.gr (A.K.); 3 Orthopaedic Surgery & Traumatology, University General Hospital “ATTIKON”, Medical School, National and Kapodistrian University of Athens, 12462 Athens, Greece; pjporthopedic@gmail.com

**Keywords:** pain, intervertebral disc, herniation, tubular discectomy, injection, alcohol

## Abstract

The purpose of this study was to retrospectively compare efficacy and safety between intradiscal injection of a gelified ethanol product and tubular discectomy in the treatment of intervertebral disk herniation. A bi-central institutional database research identified forty (40) patients suffering from symptomatic contained disc herniation. Nucleolysis Group included 20 patients [mean 50.05 ± 9.27 years-of-age (male/female 14/6–70/30%)] and Surgery Group included 20 patients [mean 48.45 ± 14.53 years-of-age, (male/female 12/8–60/40%)]. Primary outcome was overall 12-month improvement over baseline in leg pain (NVS units). Procedural technical outcomes were recorded, and adverse events were evaluated at all follow-up intervals. CIRSE classification system was used for complications’ reporting. Mean pre-operative pain score in Nucleolysis Group was 7.95 ± 0.94 reduced to 1.25 ± 1.11 at month 1 and 0.45 ± 0.75 NVS units at year 1. Mean pre-operative pain score in Surgery Group was 7.65 ± 1.13 reduced to 1.55 ± 1.79 at month 1 and 0.70 ± 1.38 NVS units at year 1. Pain decrease was statistically significant after both procedures (*p* < 0.001). There was no statistically significant difference between pain reduction in both groups (*p* = 0.347). The decrease differences of the pain effect upon general activities, sleeping, socializing, walking, and enjoying life in the follow-up period between the two groups were not statistically significant. No complications were noted in both groups. Results from the current study report that intradiscal injection of a gelified ethanol and tubular discectomy were equally effective on terms of efficacy and safety for the treatment of symptomatic lumbar intervertebral disc herniation regarding the 12-month mean leg pain improvement. Both achieved similar rapid significant clinical improvement persisting throughout follow-up period.

## 1. Introduction

Intervertebral disc herniation constitutes the most frequent reason for visits to an orthopedic or neurosurgeon and the fifth most common cause of hospitalization due to severe pain resulting in absence from work or daily activities [1]. At present, therapeutic armamentarium for symptomatic disc herniation includes conservative therapy, infiltrations, percutaneous disc decompression techniques, and surgical options. Percutaneous approaches for disc decompression include mechanical, chemical, and thermal options [2,3,4]. On the other hand, the application of endoscope contributed to minimizing invasiveness, operating time, and blood loss of surgical options [5,6,7].

When compared to conservative therapy, percutaneous decompression techniques were shown to provide significantly better and longer lasting effects; in addition, various comparative studies and systematic reviews of the literature focusing mainly on mechanical, thermal, and ozone chemical decompression showed non-inferiority when compared to surgical options [8,9,10,11,12]. Among the percutaneous decompressive techniques for the management of intervertebral disc herniation nucleolysis is a minimally invasive, image-guided technique during which a wide variety of chemical agents can be intradiscally injected aiming to gradually dehydrate and shrink the target herniated disc. Initial agent used in nucleolysis was chymopapain, which, however, was abandoned for decades mainly due to the frequent allergic reactions related to its use. Alternative agents used in nucleolysis nowadays include oxygen–ozone or a radiopaque gelified ethanol (RGE). RGE is a commercially available product the ingredients of which include ethyl alcohol, cellular derivative products, and tungsten as contrast medium for this viscous solution to be visible under image guidance; RGE is indicated for intradiscal injection in patients with symptomatic herniation [13]. Prospective and retrospective case series in the literature supported the application of Discogel and reported significant pain reduction and mobility improvement in treated patients; moreover, like other percutaneous decompression approaches, application of such a therapy does not exclude any future surgical approach in case of failure [14,15].

The purpose of the present study was to retrospectively compare efficacy (in terms of pain reduction) and safety between Discogel injection and tubular discectomy in the treatment of intervertebral disk herniation.

## 2. Materials and Methods

All patients were informed about the technique itself as well as possible benefits and complications and signed a relevant written informed consent form prior to the procedure. This bi-institutional retrospective cohort study received institutional review board approval. Clinical data were abstracted from prospectively maintained registries. The need for written informed consent for this study was waived.

A bi-central institutional database study, conducted from 1 January 2020 to 31 December 2020, identified 40 patients suffering from pain due to single intervertebral disk herniation with no neurologic deficit. All patients in the Nucleolysis Group were treated in the same center (Center 1); all patients in the Surgery Group were also treated in the same center (Center 2). All patients complained of leg pain with or without back pain and described a lancinating, burning, stabbing, or electrical sensation of pain. When leg and back pain coexisted, symptoms in the lower extremity were always of greater intensity. In each patient, symptoms were consistent to the segmental level and side of the herniation (an L3–L4 left foraminal herniation is expected to produce left L3 root neuralgia). Clinical evaluation included Laseque, Slump, and Wasermann tests. All patients included in the study had undergone electromyography testing to avoid uncertainty of the disc to be treated [4,16]. Inclusion criteria included adult patients capable of providing informed consent with symptomatic small-to-medium-sized lumbar disc herniation occupying less than one third of the spinal canal; all patients had undergone magnetic resonance imaging (MRI) showing the single hernia, nerve compression as well as potential degenerative changes of the disc including loss of height or of water content, as shown by the signal intensity of the disc. Exclusion criteria included pregnancy, significantly dehydrated intervertebral disc (“black disc”) without presence of herniation, response to a 4–6 weeks course of rigorous conservative treatment, infection, herniation occupying more than one-third of the spinal canal diameter, herniation with spinal stenosis, intervertebral disc sequestrate, neurologic deficit, spondylolisthesis, and asymptomatic patients or patients with non-correlating pain. Patients not able to undergo MRI due to contraindications or claustrophobia and patients treated with other percutaneous or surgical methods or cancer patients with metastatic disease at the level to be treated were also excluded from the current study. The diagnosis was made by an interventional radiologist with 20 years of experience and an orthopedic surgeon with 25 years of experience who identified the potential participants and verified their eligibility. All patients had undergone different conservative therapies without success. In the present study, pre-enrolment conservative therapy was not prespecified but potentially included a 4–6-week course of analgesics, anti-inflammatory drugs, muscle relaxants, and physiotherapy.

Prior to any therapeutic procedure, each patient underwent a thorough clinical examination, review of his or her medical records, and evaluation of previous imaging studies. Preprocedural imaging included lumbar spine face and lateral x rays (where the current study was performed, this is the standard examination when a patient is admitted to a hospital for low back pain and sciatica) and multiplanar MR imaging (T1-weighted, T2-weighted, and short inversion time inversion-recovery sequences at 1.5-T field strength).

**Figure 1:**
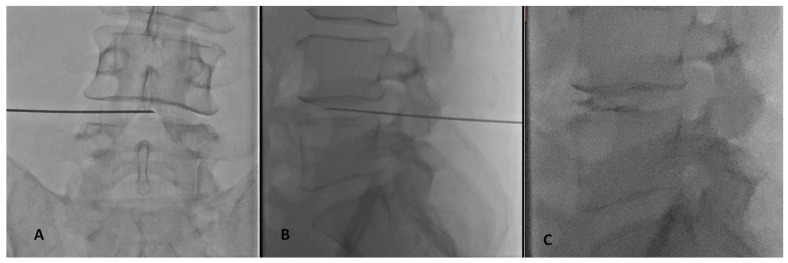
(**A**) A-P fluoroscopy view during needle placement in intradiscal Discogel injection for the treatment of an L4–L5 intervertebral disc herniation. Final position of the needle should in A-P projection in the midline between the two end plates. (**B**) Lateral fluoroscopy view during needle placement in intradiscal Discogel injection for the treatment of an L4–L5 intervertebral disc herniation. Final position of the needle should be to towards the anterior third of the intervertebral disc between the two end plates. (**C**) Lateral fluoroscopy view post intradiscal Discogel injection for the treatment of an L4–L5 intervertebral disc herniation. Discogel can be seen as radio-opaque material inside the disc.

Intervertebral disk nucleolysis was performed by intradiscal injection of Discogel (Gelscom, 8, avenue Dubna ZAC Citis F-14200 Hérouville-Saint-Clair, France) under fluoroscopic imaging guidance, local anesthesia in accordance with the Cardiovascular and Interventional Radiological Society of Europe Standards of Practice for percutaneous treatment of intervertebral disks [4]. Intravenous injection of short-term broad-spectrum antibiotic therapy including 2 g of amoxicillin/clavulanic acid or 1 g of ceftriaxone disodium was performed 30–60 min prior to the procedure. Local anesthesia was performed at the level of skin and subcutaneous fat. An 18 Gauge trocar was inserted in the intervertebral disc of interest using a posterolateral percutaneous access in all cases. The final position of the trocar was midway between vertebral endplates at the midline in postero-anterior and at the anterior third in lateral fluoroscopy views (Figure 1).

**Figure 2:**
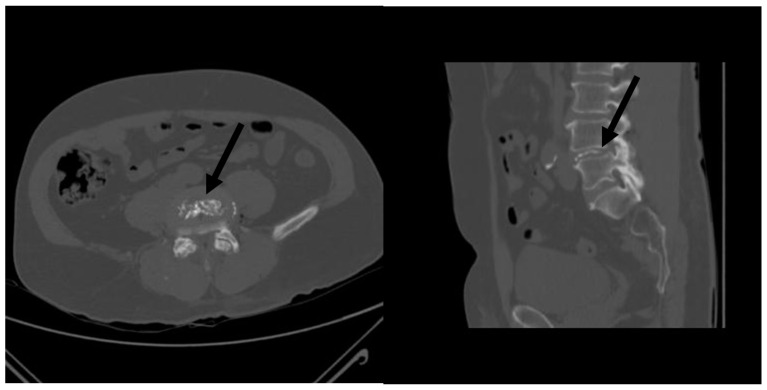
Axial and sagittal CT reconstructions performed immediately post RGE injection aiming to evaluate distribution of the product (black arrows) and rule out any potential extradiscal leakages.

In order to increase its visibility under fluoroscopy, RGE should be accurately shaken before being injected inside its container. Once ready, RGE was aspirated in syringes contained in the product kit and was slowly injected through the trocar under continuous fluoroscopic guidance to control for extradiscal leakage. The recommended dose is up to 0.8–1 mL for lumbar disks. At the end of the intradiscal injection, each patient underwent cone beam CT with 3D reconstructions in order to verify the distribution of RGE and rule out extradiscal leakages (Figure 2).

Each patient was observed for 2 h after the procedure and then discharged with a prescription for post procedure nonsteroidal anti-inflammatory drugs and muscle relaxants.

Tubular microdiscectomy was performed in the operating theater under general anesthesia and local sterility measures (including prophylactic antibiotics). Intravenous injection of short-term broad-spectrum antibiotic therapy including 2 g of amoxicillin/clavulanic acid or 1 g of ceftriaxone disodium was performed 30–60 min prior to the procedure. A limited skin incision and a narrow surgical corridor through the one side dissected paraspinal muscles and a regional drilled lamina were utilized in order the herniated disc to be removed with neurosurgical micro-instruments (microhooks, nerve hooks, disc forceps). The operation was performed only with the aid of the operative microscope (Figure 2). Each patient was hospitalized overnight after the procedure and then discharged with a prescription for analgesics, post procedure nonsteroidal anti-inflammatory drugs, and muscle relaxants.

## 3. Statistical Analysis-Outcome Measures

Clinical consultation (at 1 and 12 months) was used for post-therapeutic follow-up. We evaluated treatment response (clinical success in terms of pain reduction) as well as complication rates. Clinical success was defined as pain reduction (>4 pain score units) as recorded in the VAS pain scores. The pain score was evaluated using a brief pain inventory containing questions about pain intensity; answers were provided in terms of numeric visual scales. This pain inventory was given to the patients to be filled in the consultation prior treatment (baseline) and at 1- and 12 months post therapy. The definition of complications was assigned according to the Cardiovascular and Interventional Radiological Society of Europe (CIRSE) classification system [17].

Statistical analysis was performed using IBM SPSS Statistics for Windows, Version 26.0. Armonk, NY, USA: IBM Corp. Values are summarized and presented using mean ± standard deviation. General linear model analysis for repeated measurements was performed. Mauchly’s test of sphericity was performed and when sphericity was violated the results were analyzed using the Greenhouse–Geisser correction. A *p* value < 0.005 indicated statistical significance.

## 4. Results

**Figure 3:**
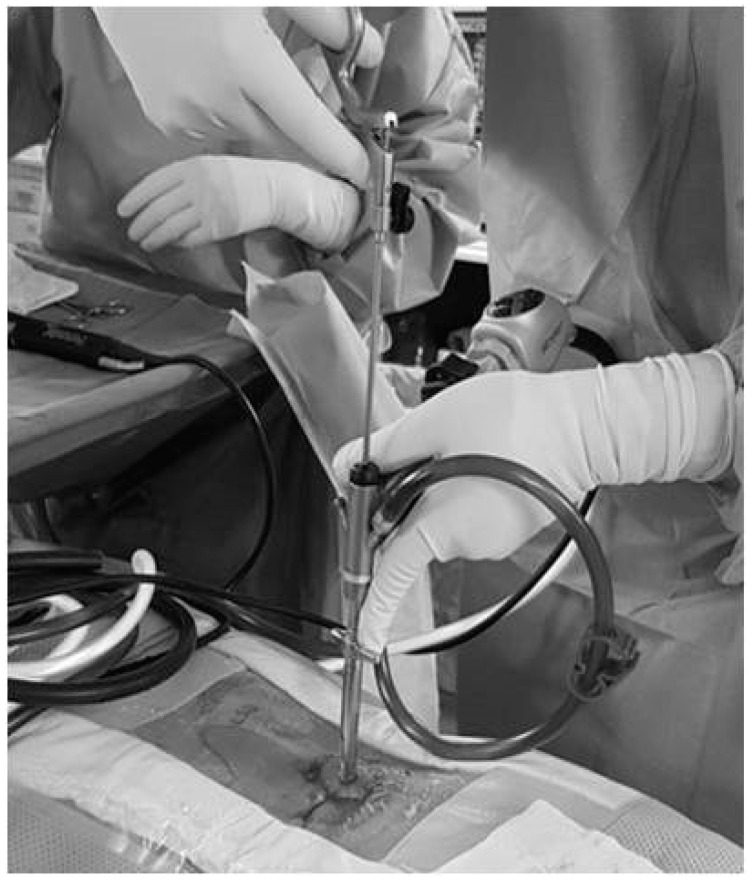
Tubular discectomy of L4–L5 intervertebral disc herniation. Both the introduction tube of the endoscope (at the surgeon’s left hand) as well as the long disc forceps for the removal of the disc fragments (at the right hand of the surgeon) are visible.

**Figure 4:**
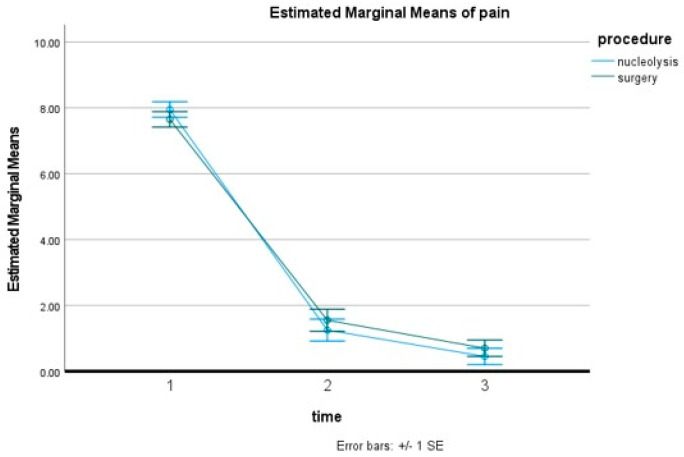
Estimated marginal means of pain scores. 1: baseline, 2: 1 month follow-up period, 3: 12 months follow-up period.

**Figure 5:**
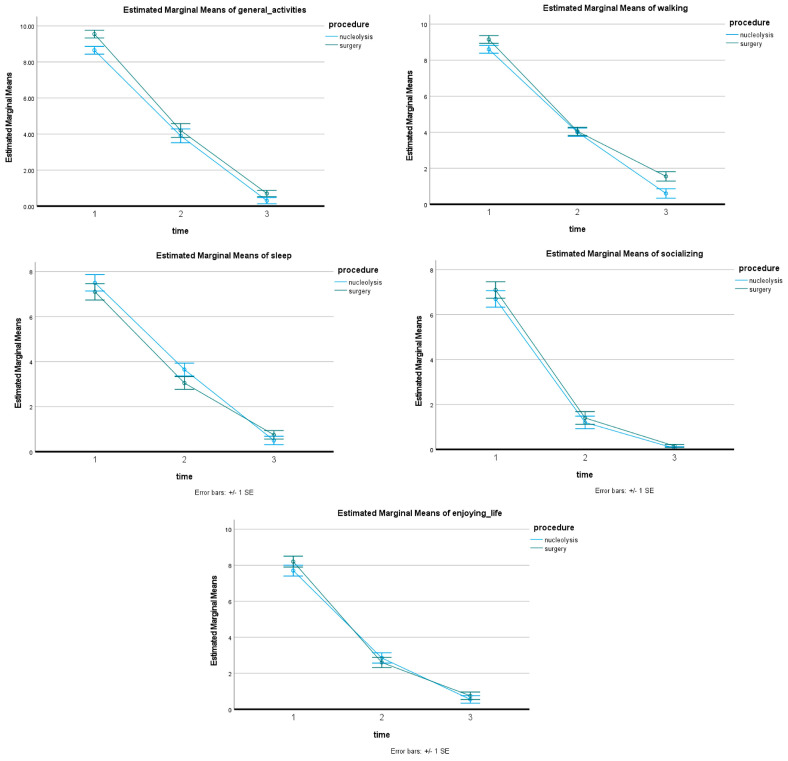
Estimated marginal means of pain decrease and its effect upon general activities, sleeping, walking, socializing, and enjoying life. 1: baseline, 2: 1 month follow-up period, 3: 12 months follow-up period.

Treatment was technically successful in all 40 patients included in the current study. the Nucleolysis Group included 20 patients [mean 50.05 ± 9.27 years-of-age (male/female 14/6–70/30%)]; treated disc levels in the Nucleolysis Group included L3–L4 (*n* = 4), L4–L5 (*n* = 10) and L5–S1 (*n* = 6). Mean pain score in the Nucleolysis Group prior to any treatment was 7.95 ± 0.94 NVS units, which was reduced to 1.25 ± 1.11 NVS units at month 1 and 0.45 ± 0.75 NVS units at year 1. All patients in the Nucleolysis Group self-reported significant pain reduction and none of them underwent other percutaneous treatments, surgical discectomy, or other conservative management including anesthesiologist medical management as an alternative for refractory and persistent symptoms. The Surgery Group included 20 patients [mean 48.45 ± 14.53 years-of-age, (male/female 12/8–60/40%). Treated disc levels in the Surgery Group included L2–L3 (*n* = 1), L3–L4 (*n* = 4), L4–L5 (*n* = 9), and L5–S1 (*n* = 6). Mean pre-operative pain score in Surgery Group was 7.65 ± 1.13 NVS units, which was reduced to 1.55 ± 1.79 NVS units at month 1 and 0.70 ± 1.38 NVS units at year 1. Pain decrease (Table 1) was statistically significant after both procedures (*p* < 0.001) (Figure 3). There was no statistically significant difference between pain reduction after surgery and after nucleolysis either at 1 or at 12 months (*p* = 0.347), (Figure 4). There was no statistically significant difference between inmprovement in general activities, walking, sleeping, socializing and enjoying life after surgery and after nucleolysis either at 1 or at 12 months (Figure 5).

In the Nucleolysis Group, the mean procedure time from patient’s entry to exit of the angiography suite was 42.55 ± 2.34 min, whilst in the Surgery Group, the mean procedure time from patient’s entry to exit of the operating theater was 95.6 ± 3.35, (*p* < 0.005). All therapeutic sessions in the Nucleolysis Group were performed as outpatient procedures. Patients in the Surgery Group were hospitalized overnight and exited the hospital the morning after the discectomy. In the Nucleolysis Group, minor extradiscal leakages (Grade 1) were depicted in the CBCT scan performed immediately post injection in 5/19 patients; all fi cases of extradiscal leakages were without any clinical significance and none of the patients reported any symptoms. There were no allergic reactions to RGE or other drugs. Transient back or lower extremity (ipsilateral to the treated side) pain was noted in 3/20 patients of the Nucleolysis Group recovering spontaneously within minutes after treatment; these temporary symptoms were attributed most probably to the trocar’s route of approach being either close to a nerve root or during its passage from the annulus fibrosus of the target intervertebral disc. No complications were noted in the Surgery Group. There were no cases of local or systemic iatrogenic infection. During the follow-up period, there were no deaths related to the procedure.

## 5. Discussion

Theron et al. introduced, for the first time, to clinical practice RGE as a market product in 2007; this specific product was designed for percutaneous intradiscal injection aiming to cause dehydration of the nucleus pulposus with subsequent shrinkage of the intervertebral disc and retraction of the herniated fragment [18]. Specifically for clinical application of RGE product in the lumbar spine, multiple case series reported significant safety and efficacy rates [18,19,20]. Theron et al. evaluated 221 patients with symptomatic intervertebral disc herniation who underwent intradiscal RGE injection, reporting 91.4% success rate and <0.5% complications rate [18]. Similarly, Stagni et al., as well as Bellini et al., reported success rates > 75% without clinically significant complications [19,20].

The present retrospective comparative study added to the growing number of case series and comparative reports showing that percutaneous decompressive approaches for the management of primary single-level intervertebral disc herniation with refractory radicular leg pain meet non-inferiority criteria and constitute comparable treatments to surgical approaches [12,18,19,20,21,22,23,24,25]. More specifically, in the present study, the comparison of pain reduction scores between percutaneous intradiscal injection of a commercially available gelified ethanol product and tubular discectomy in symptomatic patients suffering from leg pain due to single disc herniation showed no statistically significant difference (*p* = 0.347). Both nucleolysis and tubular discectomy resulted in significant pain reduction from week 1, which was sustained throughout the 12-month follow-up period (*p* < 0.005). The rapid and significant effectiveness of both intervertebral disc nucleolysis and tubular discectomy was consistent with clinical outcome improvement, as reported in numerous systematic reviews and clinical studies [13,23,24]. Similarly, the decrease in the pain effect upon general activities, sleeping, walking, socializing, and enjoying life was statistically significant in both groups without any real differences affected by the treatment arm.

Intradiscal injection of RGE during percutaneous nucleolysis seemed to work in two ways: the gelling effect engaged the herniation breach, thus preventing a greater discal excursion under axial loading, whilst ethanol contributed to reduction in local inflammatory mechanisms generated by the herniation itself [20]. The fundamental advantages of this percutaneous treatment are the cosmetic skin incision, the trivial postoperative pain and blood loss, and the shorter hospital stay in comparison to other surgical options like lumbar laminectomy and discectomy. When compared to tubular discectomy, percutaneous nucleolysis of intervertebral disc herniation by injecting intradiscally a gelified ethanol product is governed by procedural advantages; due to its minimally invasive character, the technique does not involve changes to the bony anatomy and preserves all future surgical options. All patients included in the current study tolerated well the percutaneous nucleolysis procedure; when questioned at the end of the treatment, all 20 patients reported minimal or no discomfort that was temporary and did not last more than a few minutes post trocar withdrawal. Percutaneous nucleolysis was performed as an outpatient procedure with no need for general anesthesia whilst patients were discharged earlier when compared to those undergoing tubular discectomy. In the present study, all patients in the Nucleolysis Group were discharged the same day, whilst those undergoing tubular discectomy remained in the hospital overnight. Being performed as an outpatient procedure, intradiscal gelified ethanol saves hospital beds and reduces financial costs. Although variable hospital stays were reported for lumbar microdiscectomy, the shorter hospitalization length in the current study can be attributed to the younger age of the study population who was still working and was without co-morbidities [26].

Although not shown in the current study (due to its small patient sample), another advantage for the patients is the lower complication rate of percutaneous approaches when compared to the 7.6% of surgical approaches [27]. No clinically significant complications were recorded in the present study. Minor extradiscal leakages of Discogel were noted in the Discogel Group in 5/19 patients without any need for clinical management; all noted extra-discal leakages were related to reflux during the removal of the trocar. The safety of both Discogel injection in the intervertebral disc and tubular discectomy was consistent with the reported rates of numerous systematic reviews and clinical studies [13,23,24,25,28,29]. High-quality sophisticated imaging guidance with digital rotational angiography suite contributed to easier trocar insertion whilst clearly improving visibility and augmenting safety of intradiscal injections; state-of the art angiography suites provide significantly better visibility and guidance options when compared to c-arm X-ray machines. Continuous fluoroscopic monitoring should be used during Discogel injection to minimize risk of leakage, whilst the provided syringes included in the kit allow for controlled volumetric delivery [2,4]. Although percutaneous nucleolysis of symptomatic herniated intervertebral disc can be performed under CT or CT—fluoroscopy guidance, advantages of angiography suite and use of fluoroscopy include shorter duration, reduction in radiation dose, and real time monitoring during injection of RGE, which is a pre-requisite for minimizing extra-discal leakages of the product [2,4]. Like previous comparative trials, blinding of the physicians or patients on whom treatments were performed was not possible [12].

Local anesthesia was performed only at the skin surface, aiming to avoid any potential interference of anesthetic application at the nerve level. To perform a valid assessment of therapeutic effectiveness, the current study focused upon proper selection of patients with the best chance for a successful procedure; this is also essential not only for decision-making in everyday clinical practice. Since magnetic resonance imaging is considered as the gold standard for the diagnosis of intervertebral disc herniation, all patients included in the current study were evaluated for rigorous quantitative MRI criteria that were used to select candidates eligible for the study [30,31]. These criteria (which also corresponded to lumbar degenerative discs) included nerve compression due to disc herniation and loss of signal intensity [30]. In addition to the imaging criteria, a clinical assessment and dermatologic distribution of pain consistent with the radiologic findings was required. The presence of osteophytes and an intervertebral disc with significantly reduced height-increased technical difficulty, rendering trocar insertion more challenging, especially at L5–S1 disc level. Similar to Bellini et al., the current study significantly dehydrated else called “black” discs with grade V according to the Pfirman classification were considered am exclusion criterion for the following reasons: there is no distinction between nucleus and annulus of the disc target, and they are related with reduced positive prognosis due to these severe morphostructural changes [20]. Furthermore, the severe lack of water content is directly correlated the degree of the gelling effect produced by RGE presence. Free fragments separated from the intervertebral disc (sequestrum) were also an exclusion criterion: percutaneous decompression techniques including nucleolysis were based on the Hijicata theory first stated in the 1970s, highlighting that, in the closed space of intervertebral disc, any minor volume change results in significant pressure changes. Intervertebral disc free fragments by default lost the communication to the mother disc and percutaneous decompression techniques at the level of nucleus pulposus have no effect upon them.

There were several limitations of the present study apart from its retrospective character and the short follow-up that should be considered, and which could introduce selection and results’ biases; the small sample size along with the mixed levels of the treated disc hernias rendered difficult the evaluation of site-specific complications. No data regarding function improvement through specific scales were recorded; however, such questions were included and evaluated from the questionnaire used in the current study. This study did not provide a comparison with open surgery, nevertheless, our findings suggest that minimally invasive percutaneous or endoscopic approaches constitute a safe and effective technique for the management of intervertebral disc herniation. Discography was not performed to avoid the limited visualization of the RGE by the injected iodinated contrast medium and the immediate gelling effect that occurs when the product meets any fluid increasing the risk of trocar occlusion. Although technically two or three levels can be treated in the same session, only single-level intervertebral disc herniations were treated in the current study.

## 6. Conclusions

The results of the current study showed that intradiscal injection of a gelified ethanol and tubular discectomy were equally effective in terms of efficacy and safety for the treatment of symptomatic lumbar intervertebral disc herniation regarding the 12-month mean leg pain improvement. Both treatment arms achieved similar rapid significant clinical improvement, which persisted throughout the follow-up period.

## Figures and Tables

**Table 1:** Patient demographics.

Pain	Procedure	Mean	Std. Deviation	N
before	Nucleolysis	7.9500	0.94451	20
	Surgery	7.6500	1.13671	20
	Total	7.8000	1.04268	40
month 1	Nucleolysis	1.2500	1.11803	20
	Surgery	1.5500	1.79106	20
	Total	1.4000	1.48151	40
month 12	Nucleolysis	0.45	0.759	20
	Surgery	0.70	1.380	20
	Total	0.58	1.107	40

## Data Availability

The data presented in this study are available on request from the corresponding author. The data are not publicly available at the moment since the present study is the basis for the PhD thesis of the author C.G.
